# *In silico* discovery of SARS-CoV-2 main protease inhibitors from the carboline and quinoline database

**DOI:** 10.2217/fvl-2021-0099

**Published:** 2021-07-20

**Authors:** Eldar Muhtar, Mengyang Wang, Haimei Zhu

**Affiliations:** ^1^Beijing Area Major Laboratory of Peptide & Small Molecular Drugs, Engineering Research Center of Endogenous Prophylactic of Ministry of Education of China, Beijing Laboratory of Biomedical Materials, College of Pharmaceutical Sciences of Capital Medical University, Beijing, 100069, China

**Keywords:** carboline, dPCA, main protease, MD simulation, MM-PBSA, SARS-CoV-2, virtual screening

## Abstract

**Aim:** SARS-CoV-2 caused more than 3.8 million deaths according to the WHO. In this urgent circumstance, we aimed at screening out potential inhibitors targeting the main protease of SARS-CoV-2. **Materials & methods:** An in-house carboline and quinoline database including carboline, quinoline and their derivatives was established. A virtual screening in carboline and quinoline database, 50 ns molecular dynamics simulations and molecular mechanics Poisson−Boltzmann surface area calculations were carried out. **Results:** The top 12 molecules were screened out preliminarily. The molecular mechanics Poisson−Boltzmann surface area ranking showed that p59_7m, p12_7e, p59_7k stood out with the lowest binding energies of -24.20, -17.98, -17.67 kcal/mol, respectively. **Conclusion:** The study provides powerful *in silico* results that indicate the selected molecules are valuable for further evaluation as SARS-CoV-2 main protease inhibitors.

SARS-CoV-2 has spread all over the world, led to SARS and caused more than 179 million infections and 3.8 million deaths according to the WHO [1]. SARS-CoV-2 manifests higher transmissibility and lower mortality compared with SARS-CoV. SARS-CoV-2 shows efficient intrafamilial spread [2]. Although, several vaccines have been approved by the WHO such as BBIBP-CorV and CoronaVac [3], the pain and fever occur after treatment of some vaccines. The protective efficacy of the vaccines still need to be improved. Vaccines can help to prevent infection and severe symptoms caused by SARS-CoV-2. However, for patients who have been infected with the virus, drugs are still in need. Remdesivir is the first drug approved by the US FDA for the treatment of SARS-CoV-2 [4]. Remdesivir targets RNA polymerase to inhibit viral replication. However, main protease (M^pro^) is also one of the most important enzymes in the life cycle of virus. M^pro^ of SARS-CoV-2 is a crucial enzyme of coronaviruses and has a pivotal role in mediating the viral maturation [5]. Besides, M^pro^ is most abundant in the viral surface and is believed to be the crucial organizer in the coronavirus assembly [6], making it an arresting drug target for SARS-CoV-2. The protein crystal (Protein Data Bank [PDB] code: 6LU7) contains ligand N3 which help us to define the active site pocket of M^pro^ of SARS-CoV-2 [7]. Docking is useful in virtual screening of small molecule databases and predicting the structures and functions of biomolecular complexes. Molecular dynamics (MD) simulations can give a dynamic image that obtained from the molecular docking [8]. Moreover, the molecular mechanics Poisson−Boltzmann surface area (MM-PBSA) method provides a more accurate calculation of the binding energy. In previous studies, molecules in public databases, repurposed approved drugs or molecules in natural products were obtained for *in silico* screening targeting M^pro^ [9–14]. We established an in-house database, carboline and quinoline database (CQDB) that included both carboline and quinoline molecules in this study. Since isoquinoline, quinoline, β-carboline and their derivatives show the powerful antiviral bioactivity [15,16]. Based on the small molecule database, we attend to use computational approaches mentioned above to find potential molecules for the treatment of SARS-CoV-2 (Figure 1).

**Figure 1.**
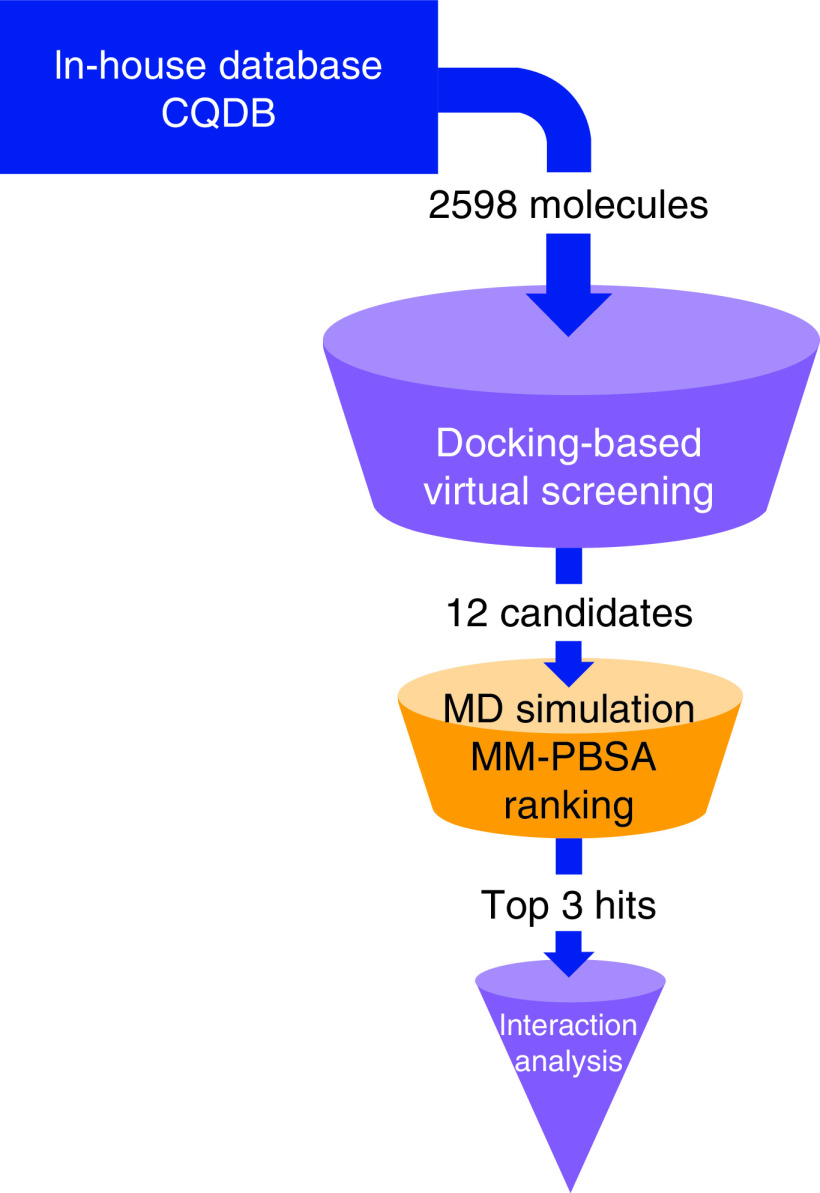
Workflow for the discovery of potential inhibitor against main protease *in silico*. CQDB: Carboline and quinoline database; MD: Molecular dynamics; MM-PBSA: Molecular mechanics Poisson−Boltzmann surface area.

## Materials & methods

### Dataset

For this study, we established a small molecule database named “CQDB”. CQDB includes 2598 carboline and quinoline derivatives without duplicated molecules. 1117 molecules were synthesized and patented in our previous work. Another 1481 molecules were obtained from the publications of other pharmaceutical researches. 2D structures of these molecules were sketched in ChemBioDraw and converted to 3D structures with Open Babel v3.1.0 as ligands [17]. Afterward, optimizations were done in 250 steps of a steepest-descent geometry optimization with the MMFF94 forcefield in Open Babel.

### Protein & ligand setup

The crystal structure of SARS-CoV-2 M^pro^ (PDB code: 6LU7) was obtained from the PDB with a resolution of 2.16 angstrom. The protease consisted of one chain with 306 amino acids. 6LU7 was prepared at a pH level of 7.4 for protonation using the ‘prepare protein’ protocol in BIOVIA Discovery Studio 2016 (Dassault Systèmes, Vélizy-Villacoublay, France). The protonation state of crucial residuals such as HIS 41, HIS 164 and GLU 166 in binding-site was checked carefully again. The solvent was stripped off. Ligands were obtained from the CQDB database.

### Docking-based virtual screening

Docking-based virtual screening, which is one of the most promising methods *in silico* for the drug-like molecule discovery, is useful to predict the best interaction state between a ligand and a protein. AutoDock Vina (Scripps Research, CA, USA) was chosen to perform the virtual screening. Since AutoDock Vina provides the maximum accuracy and the minimum computer time, which refers to the empirical and knowledge-based scoring functions [18]. Ligands were assigned with the gasteiger charges. The grid box size in three dimensions was 40 × 40 × 40 Å with a center coordinate of -7.857, 11.856 and 67.687, which was the center of the ligand N3 in the crystal structure. The exhaustiveness of the global search was increased to 12. Based on the binding affinity ranking, the top 12 molecules that satisfied a threshold (ΔG ≤−9.8 kcal/mol) were screened out for more detailed analysis.

### Strategy of docking & selection of promising configuration

In the docking analysis, a theoretical method to identify the appropriate configuration of ligand in enzyme active-site is very important [19,20]. To evaluate the docking and selection strategy, N3 was fetched out from the crystal structure and redocked into M^pro^ as a reference. Covalent docking was performed using AutoDock 4.2.6 [21]. The grid box dimensions were 40 × 40 × 40 Å. The grid spacing was set as 0.375 Å. Lamarkian genetic algorithm was utilized to find the appropriate configurations of ligands. The global optimization was performed with parameters of 300 randomly positioned individuals. The maximum number of energy evaluations was enhanced to 2.5 × 10^7^, and the maximum number of generations in lamarkian genetic algorithm was enhanced to 2.7 × 10^5^. The Solis and Wets local search was executed with a maximum number of 3000. During docking experiments 200 runs were carried out. The resulted 200 conformations of each were ranked by the lowest binding energy and clustered with an all-atom root mean square deviation tolerance of 2.0 Å. The lowest binding energy, the population of the configuration in the cluster analysis and the proper binding mode were considered comprehensively for selecting the promising configuration of N3. Subsequently, the top 12 molecules were redocked toward the M^pro^ in AutoDock 4.2.6 to select the promising configurations using the same selection strategy. The selected promising configurations of the 12 molecules were submitted as the start configurations for the MD simulations.

### MD simulation analysis

MD simulation is a decision-making procedure for the evaluation of complex stability [22]. It is useful in the investigation of the dynamic behavior at an atomic level of biological systems, which is hard to process in the laboratory [23]. In the present study, the most promising configurations of the 12 M^pro^–ligand complexes were chosen as the starting point for MD simulations. MD simulations for 12 M^pro^–ligand complexes and an apo form of the M^pro^ were performed on a 50 ns time scale. The CHARMM General Force Field web-based tool (https://cgenff.umaryland.edu/) was used to generate ligand parameter files. Charge of ligands was assigned by the extended bond–charge increment scheme [24]. Gromacs 2020.1 was utilized to perform the simulation with the CHARMM36 all-atom force field [25,26]. All of the 13 systems were solvated with an extended simple point charge model (SPC/E) and neutralized via adding Na^+^ ions [27]. In the following process, the energy of the system was minimized by the steepest descent algorithm at a threshold of 1000 kJ·mol^-^^1^·nm^-^^1^. Then two-part equilibration, conserved moles, volume and temperature (NVT) and conserved moles, pressure and temperature (NPT) ensembles were done for 0.5 ns. Long-range electrostatics was calculated by the particle Mesh Ewald method [28]. About 50 ns MD simulations were performed at a time step of 2 fs. Then root mean square deviation (RMSD), root mean square fluctuation (RMSF), radius of gyration (Rg) and the number of hydrogen bonds were calculated to analyze the MD trajectories in GROMACS utilities.

### MM-PBSA combined with MD

MM-PBSA is used in study of biomolecular interactions and the computational drug design. MM-PBSA binding energy of chosen molecules was calculated via g_mmpbsa [29]. This tool calculates the enthalpic components of the MM-PBSA interaction using GROMACS and the APBS packages. The total binding free energy is calculated as follows: $\Delta G_{binding}=G_{complex}-(G_{protein}+G_{ligand})$

### Dihedral angle principal component analysis

The principal component analysis (PCA) method was used to calculate eigenvectors and eigenvalues and their projection along with the first two principal components during MD simulation. The dihedral angle principal component analysis (dPCA), which is based on the Gromacs protocol, uses backbone dihedral angles to analyze while PCA uses Cartesian coordinates [25]. dPCA can readily be characterized by the corresponding conformational changes of peptides in a protein [30]. dPCA was calculated from the MD backbone trajectories. Via diagonalizing the matrix, a bunch of eigenvectors and eigenvalues were generated and plotted in the 2D projection to evaluate the motion of trajectory.

## Results

### Virtual screening

To evaluate the potential of molecules in our database to become inhibitors of M^pro^, AutoDock Vina was used to screen in the database and rank molecules according to their binding affinities. The name, structure and molecular weight of the top 12 molecules are listed in Table 1.

**Table 1.** The name, structure, molecular weight and binding free energies of the top 12 molecules virtually screened from in-house carboline and quinoline database.

Rank	Name	Structure	Molecular weight	ΔG from AutoDock (kcal/mol)	ΔG from MM-PBSA (kcal/mol)
1	p59_7m		789.84	-8.39	-24.20
2	p63_9h		462.48	-8.60	-17.98
3	p12_7e		544.39	-9.10	-17.67
4	p59_7k		794.26	−8.70	−17.51
5	22_7b		881.95	-5.55	-16.44
6	p63_9l		490.52	-8.22	-16.07
7	p63_9m		448.46	-8.28	-14.55
8	506_4Rd		525.61	-7.10	-13.48
9	p63_9i		458.52	-8.92	-12.94
10	p63_9j		445.48	-8.00	-12.24
11	310_5g		559.62	-7.82	-11.30
12	p80_7r		518.96	-9.34	-7.90

Binding free energies calculated with both AutoDock and MM-PBSA. The top 12 molecules ranked by MM-PBSA binding energy.

MM-PBSA: Molecular mechanics Poisson−Boltzmann surface area.

### Selection of proper configurations of referenced inhibitor

**Figure 2.**
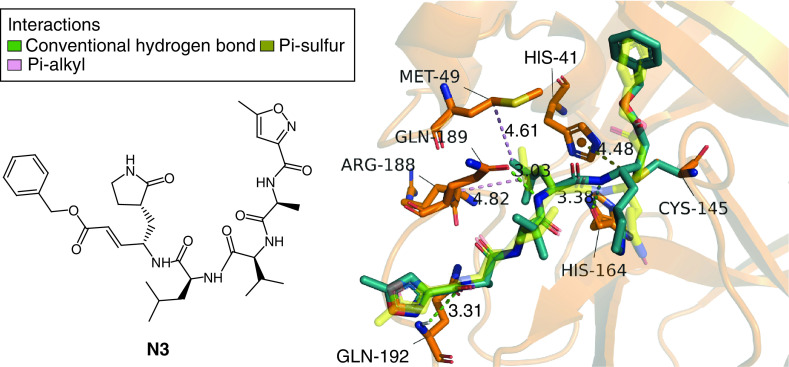
Molecular interactions between main protease (orange ribbon) and docked main protease inhibitor N3 from crystal structure 6LU7 (deep teal stick). Crystal structure of M^pro^ inhibitor N3 shown as yellow transparent stick model.

The proper docked configuration of N3 was selected and shown in Figure 2. The selected configuration of N3 is well overlapped with N3 in the crystal structure. The binding free energy of the selected configuration of N3 to the M^pro^ is -15.40 kcal/mol in AutoDock 4.2.6. Compared with the crystal N3, the selected configuration of N3 forms similar molecular interactions with the M^pro^, including conventional hydrogen bonds with GLY 143, HIS 164, GLN 189 and GLN 192, hydrophobic interactions such as Pi-Sulfur, Pi-Alkyl with MET 49 and HIS 41 and ARG 188. The result suggests that the selection strategy of proper configuration of the ligand is appropriate.

Using the same docking and selection strategy, the top 12 molecules were redocked with AutoDock 4.2.6. The most promising configurations of the 12 molecules are selected and the lowest binding energies calculated by AutoDock 4.2.6 are listed in Table 1.

### Reranking with MM-PBSA binding energy

**Figure 3.**
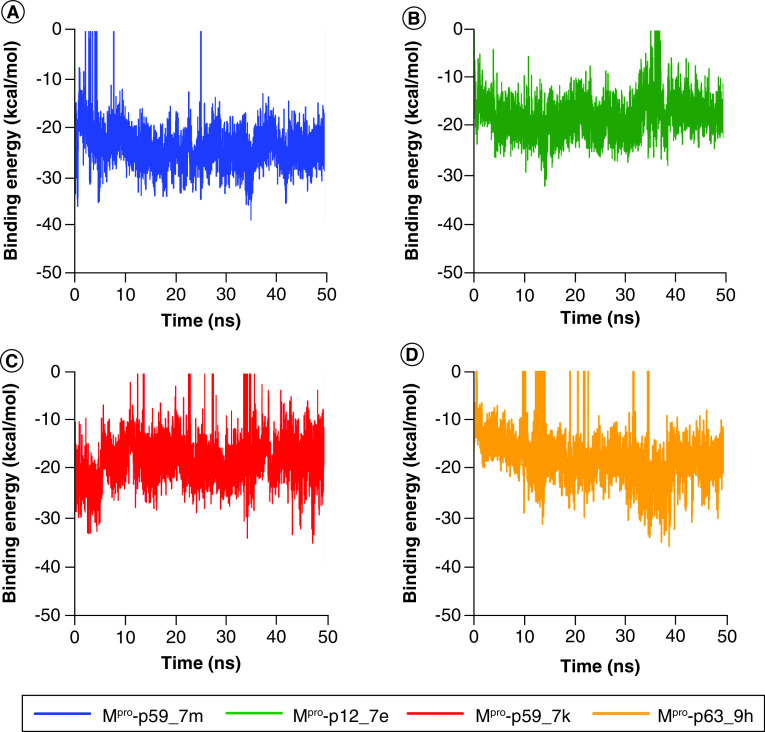
The molecular mechanics Poisson−Boltzmann surface area binding energies over 50 ns simulations of the top four ranked molecules. M^pro^: Main protease.

The top 12 molecules were reranked according to the MM-PBSA binding energies shown in Table 1. The top four molecules are p59_7m, p63_9h, p12_7e and p59_7k. Figures 3A–D shows the fluctuation of MM-PBSA binding energies of the top four molecules during 50 ns MD simulations. The MM-PBSA binding energy of the top four molecules fluctuated stably during 50 ns. Table 2 shows contributions in MM-PBSA binding energy of the top four molecules.

**Table 2.** Contributions in molecular mechanics Poisson−Boltzmann surface area binding energies of the top four molecules.

Name	ΔE_ele_	ΔE_vdw_	ΔG_np_	ΔG_p_	ΔG_bind_
p59_7m	-13.29	-51.16	-5.83	46.07	-24.20
p63_9h	-4.21	-34.70	-3.69	24.62	-17.98
p12_7e	-7.36	-45.31	-4.68	39.68	-17.67
p59_7k	-14.93	-51.48	-6.05	54.96	-17.51

The unit of all parameters is kcal/mol.

ΔE_ele: _Electrostatic energy; ΔE_vdw:_ Van der Waal energy; ΔG_np:_None polar solvation energy (solvent-accessible surface area energy); ΔG_p:_ Polar solvation energy; ΔG_bind_: Binding energy.

### Binding stability evaluations during MD simulation

**Figure 4.**
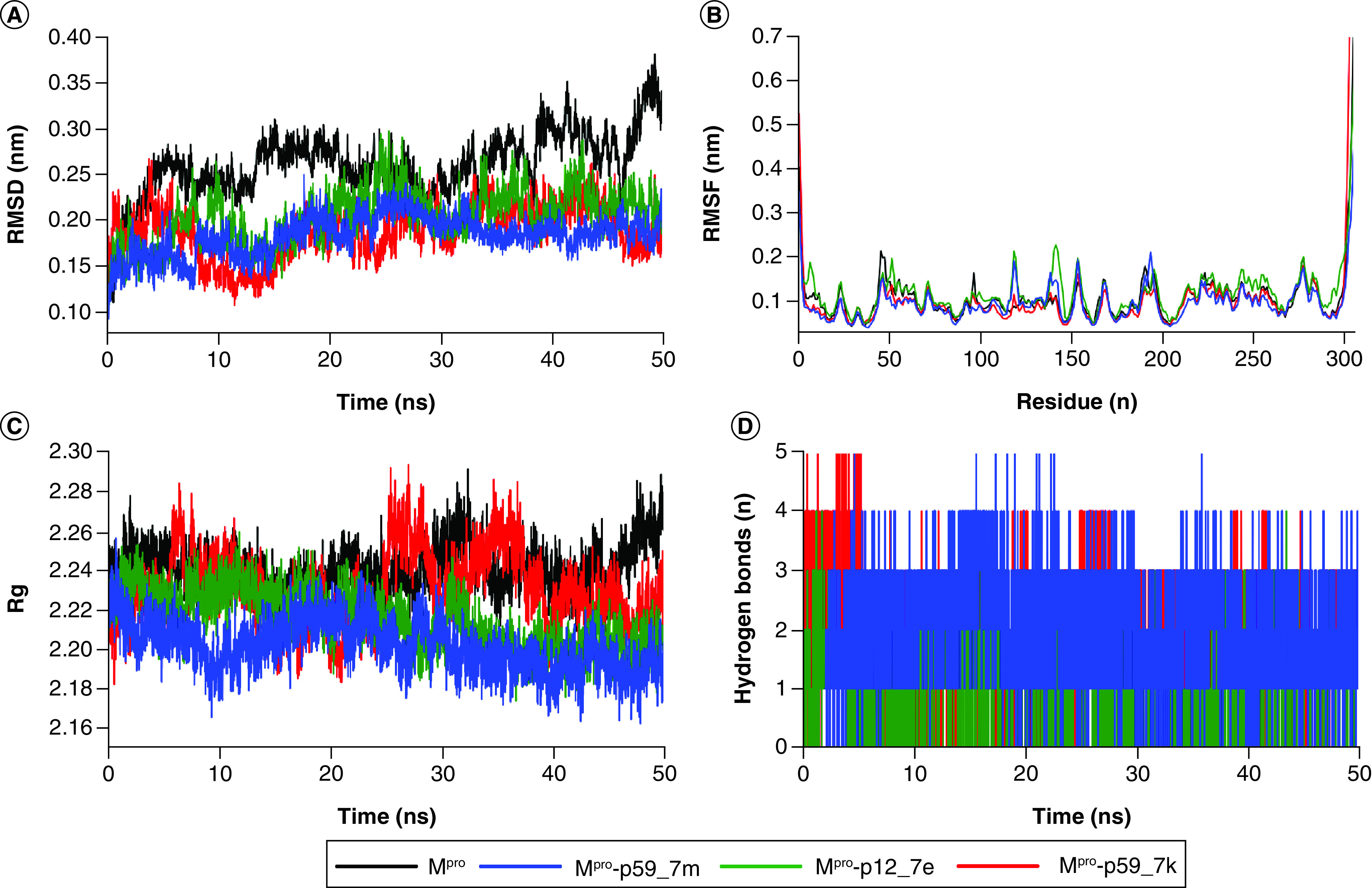
The molecular dynamics simulation analysis of apo SARS-CoV-2 main protease and three main protease–ligand complexes. **(A)** Root mean square deviation values of the M^pro^ backbone over time. **(B)** Root mean square fluctuation values of the M^pro^ backbone during 50 ns simulations. **(C)** Radius of gyration of the main protease over time. **(D)** Hydrogen bond number between main protease and ligands over time. M^pro^: Main protease; Rg: Radius of gyration; RMSD: Root mean square deviation; RMSF: Root mean square fluctuation.

To evaluate the binding stability of the top 12 molecules at the binding site of M^pro^, the protein backbone RMSD, RMSF, the Rg and the number of hydrogen bonds, the protein dihedral principal component (dPCA) and the ligand binding mode during 50 ns MD simulations were carefully inspected. It was found that p63_9h exhibited unstable binding mode at the active site of the M^pro^. Thus, p59_7m, p12_7e and p59_7k were suggested as the top three molecules with the potential of inhibiting M^pro^. The average protein backbone RMSD of apo form is 0.26 nm while holo forms with p59_7m, p12_7e, p59_7k are 0.18, 0.20, 0.19 nm, respectively (Figure 4A). This result suggests the reduction of overall protein flexibility upon binding of the three selected molecules. The protein backbone RMSF shown in Figure 4B represents lower fluctuations of protein residues during MD simulation upon binding p59_7m and p59_7k than binding p12_7e. The Rg shown in Figure 4C exhibits more compact protein–ligand complex during MD simulation upon binding p59_7m and p12_7e than binding p59_7k. The number of hydrogen bonds reflects one of the crucial interactions between the protein and the corresponding ligand. As seen in Figure 4D, average number of hydrogen bonds upon binding of p59_7m, p12_7e, p59_7k are 2.0, 1.2 and 1.8, respectively. This suggests p59_7m forms stronger hydrogen bond interactions than p59_7k and p12_7e.

### Dihedral principal component analysis

**Figure 5.**
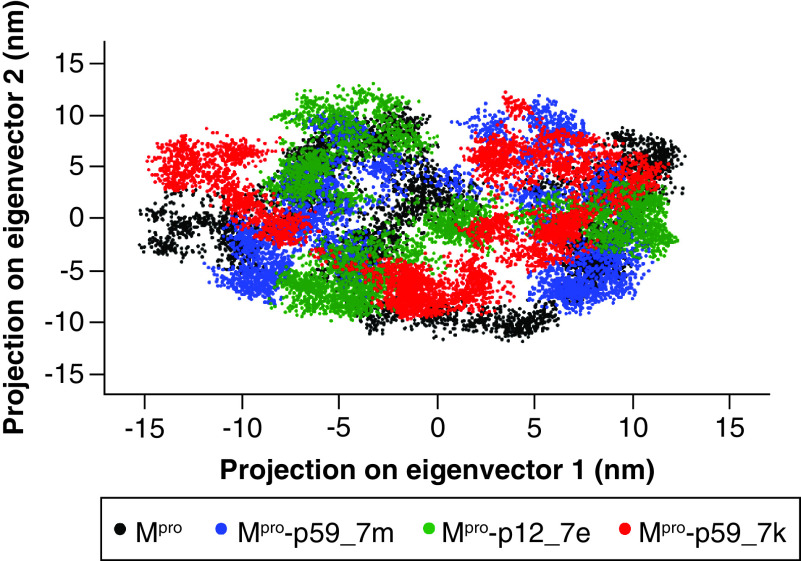
2D projection of motion of trajectory of apo SARS-CoV-2 main protease and three main protease–ligand complexes over the first two principal components. M^pro^: Main protease.

The dihedral principal component analysis (dPCA) method was employed to reveal the dynamical behavior in the space of SARS-CoV-2 M^pro^ when combined with the top three molecules. The first two principal components were selected to analyze the projection of apo form phase space and holo form phase spaces with top three molecules during the 50 ns MD simulations. Figure 5 clearly shows that apo protein and the M^pro^-p59_7k complex covered a larger region of phase space while the M^pro^-p59_7m complex and M^pro^-p12_7e complex covered smaller ones. The result suggests that binding of p59_7m and p12_7e in the active site limits large dynamic behaviors of SARS CoV-2 M^pro^, which is in accordance with the Rg results.

### Protein–ligand interaction analysis

**Figure 6.**
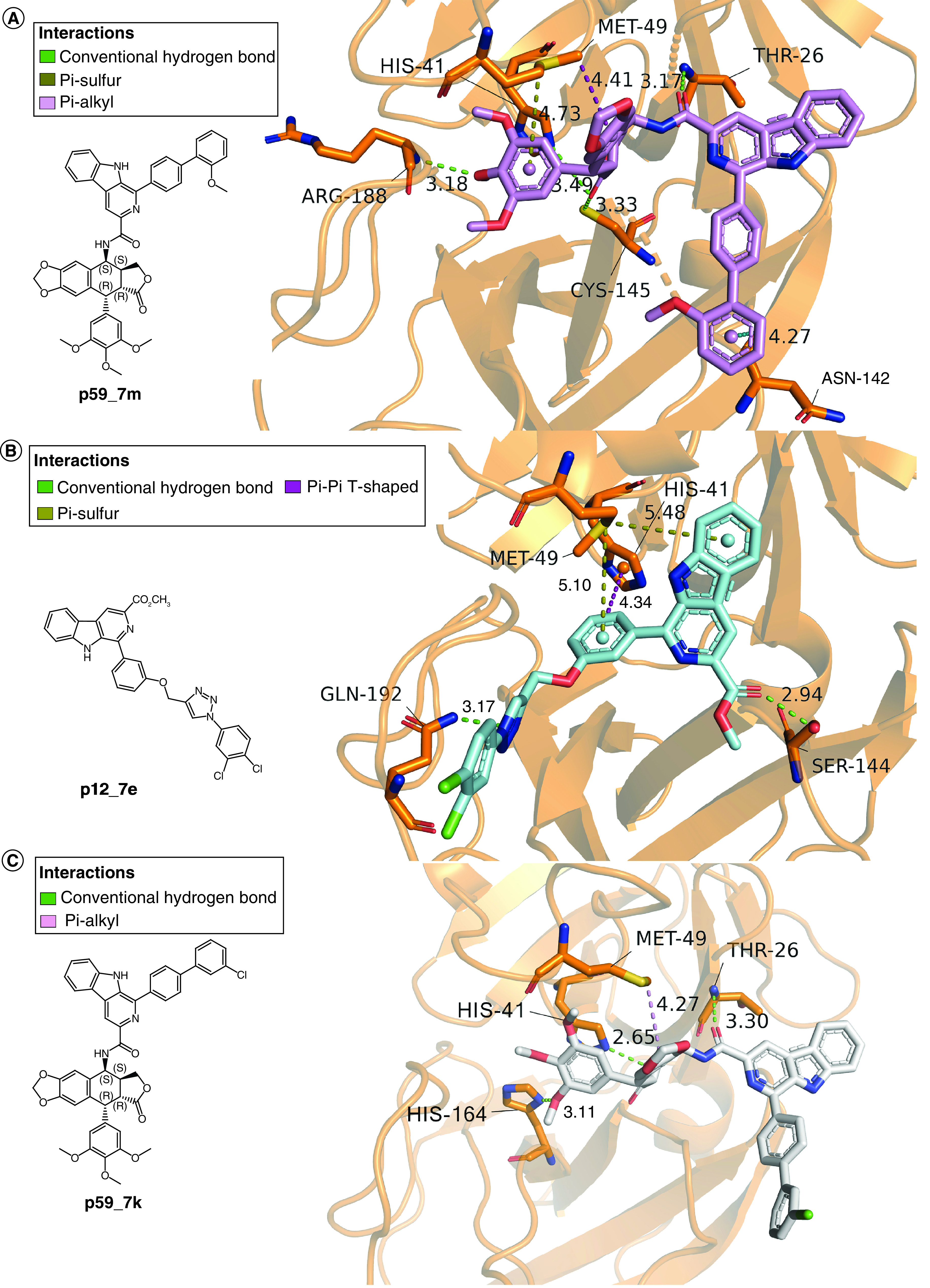
Molecular interactions between main protease and the top three molecules in the last snapshots of molecular dynamics simulations. **(A)** p59_7m in the active site of main protease (M^pro^). **(B)** p12_7e in the active site of M^pro^. **(C)** p59_7k in the active site of M^pro^.

The 3D interactions of the top three molecules with M^pro^ in the last snapshot from MD simulation are shown in Figures 6A–C. Figure 6A reveals p59_7m forms four conventional hydrogen bonds with THR 26, HIS 41, CYS 145, ARG 188 in range: 3.17–3.49 Å. While p12_7e (Figure 6B) and p59_7k (Figure 6C) forms three and two conventional hydrogen bonds with M^pro^, respectively. Besides, p59_7m forms hydrophobic interactions with MET 49 and ASN 142.

## Discussion

SARS-CoV-2 invades human respiratory system, spreads rapidly and causes a severe health crisis all over the world. Vaccines can help to prevent infection and severe symptoms caused by SARS-CoV-2. However, drugs treating SARS-CoV-2 infection are still in urgent need. The RNA polymerase inhibitor, remdesivir, is the only drug approved by FDA up to now. Nowadays, The M^pro^ of SARS-CoV-2 has become one of the most promising targets for new drugs. The M^pro^ inhibitor PF-07321332 from Pfizer has entered a Phase I clinical study [31]. Carboline and quinoline molecules were reported to possess powerful antiviral bioactivities. An in-house database CQDB containing 2598 carboline and quinoline molecules was established for the discovery of M^pro^ inhibitors. Docking-based virtual screening and following MD simulations revealed three potential M^pro^ inhibitors p59_7m, p12_7e and p59_7k. Among the three molecules, p59_7m exhibits the lowest binding free energy of -24.20 kcal/mol. p59_7m forms the most extensive and stable hydrogen bond and hydrophobic interactions with M^pro^ active site residues THR 26, HIS 41, MET 49, ASN 142, CYS 145 and ARG 188, which are shown to interact with peptidomimetic inhibitors in the crystal structures [7,32]. Different from the peptidomimetic inhibitors in the crystal structures which spans S1′ to S4 pocket, p59_7m binds at S2′ to S2 pocket. The carboline derivative p59_7m is worthy of further investigation.

SARS-CoV-2 may predispose to both venous and arterial thromboembolic disease due to excessive inflammation, hypoxia, immobilization and diffuse intravascular coagulation [33]. Previous works indicate carboline and quinoline molecules decrease both arterial and venous thrombus *in vivo* [34,35]. Also, carboline and quinoline molecules may have anti-inflammatory activity [34,36]. Therefore, the selected carboline derivatives may also have the potential of reducing thrombus and inflammatory syndromes of SARS-CoV-2 in the future perspective.

## Conclusion

Among 2598 molecules in the database we established, the top 12 molecules were selected through the docking-based virtual screening using AutoDock Vina. Then MD simulations were performed on the apo form and the 12 docked complexes of SARS-CoV2 M^pro^ and molecules to confirm their system stabilities. Based on the MD simulations, binding affinities of the 12 molecules with SARS-CoV-2 M^pro^ were calculated using MM-PBSA method. The top three molecules p59_7m, p12_7e and p59_7k bind SARS-CoV-2 M^pro^ with the lowest MM-PBSA binding free energies of -24.20, -17.98, -17.67 kcal/mol, respectively. They form extensive hydrogen bonds with the active site residues and obviously decreased the flexibility of SARS-CoV-2 M^pro^. The selected three molecules are worthy of further bioactivity studies against SARS-CoV-2 M^pro^. This result also encourages further exploration of bioactive carboline and quinoline derivatives against SARS-CoV-2.

## Future perspective

Previous works indicate carboline and quinoline molecules decrease both arterial and venous thrombus *in vivo*. Also, carboline and quinoline molecules have the anti-inflammatory activity in vivo from the previous research. Carboline, quinoline and their derivatives have the potential to be explored as antiviral molecules with anti-thrombosis and anti-inflammatory activities in the future.

**Summary points**

- SARS-CoV-2 causes more than 3.8 million deaths according to the WHO.
- Drugs treating SARS-CoV-2 infection are in urgent need.
- The main protease (M^pro^) of SARS-CoV-2 has become one of the most promising targets for new drugs.
- Carboline and quinoline molecules possess powerful antiviral bioactivities.
- An in-house database carboline and quinoline database containing 2598 carboline and quinoline molecules was established for the discovery of M^pro^ inhibitors.
- Docking-based virtual screening and following molecular dynamics simulations revealed three potential M^pro^ inhibitors p59_7m, p12_7e and p59_7k.
- Among the three molecules, p59_7m exhibits the lowest binding free energy of -24.20 kcal/mol.
- Similar to the peptidomimetic inhibitors in the crystal structures, p59_7m forms extensive and stable hydrogen bonds and hydrophobic interactions with M^pro^ active site residues THR 26, HIS 41, MET 49, ASN 142, CYS 145 and ARG 188.
- The carboline derivative p59_7m is worthy of further investigation of anti-SARS-CoV-2 activity.
